# A systems biology view of blood vessel growth and remodelling

**DOI:** 10.1111/jcmm.12164

**Published:** 2013-11-17

**Authors:** Elizabeth A Logsdon, Stacey D Finley, Aleksander S Popel, Feilim Mac Gabhann

**Affiliations:** a Institute for Computational Medicine and Department of Biomedical Engineering, Johns Hopkins UniversityBaltimore, MD, USA; b Department of Biomedical Engineering, Johns Hopkins University School of MedicineBaltimore, MD, USA

**Keywords:** angiogenesis, computational model, mathematical model, systems biology, multi-scale modelling

## Abstract

Blood travels throughout the body in an extensive network of vessels – arteries, veins and capillaries. This vascular network is not static, but instead dynamically remodels in response to stimuli from cells in the nearby tissue. In particular, the smallest vessels – arterioles, venules and capillaries – can be extended, expanded or pruned, in response to exercise, ischaemic events, pharmacological interventions, or other physiological and pathophysiological events. In this review, we describe the multi-step morphogenic process of angiogenesis – the sprouting of new blood vessels – and the stability of vascular networks *in vivo*. In particular, we review the known interactions between endothelial cells and the various blood cells and plasma components they convey. We describe progress that has been made in applying computational modelling, quantitative biology and high-throughput experimentation to the angiogenesis process.

- Introduction
- Systems biology approaches to understand the multi-step process of angiogenesis
  – Angiogenic stimuli
  – Sprouting
  – Elongation and branching
  – Tubulogenesis, lumen formation and anastomosis
  – Stabilization/regression
  – Quantitative high-throughput experimental approaches
- Application of systems biology approaches to therapeutic regulation of angiogenesis
  – Targeting angiogenic stimuli
  – Targeting sprouting
  – Targeting elongation and branching
  – Targeting tubulogenesis and anastomosis
  – Targeting stabilization/regression
- Conclusion and future work

## Introduction

Blood vessels provide the highways for blood trafficking including the delivery of oxygen, inflammatory and progenitor cells, as well as the removal of waste products. Without regulation of vascular highway growth, maintenance and regression, components of the bloodstream would be unable to fulfil their physiological roles and ensure organism homeostasis. Blood vessels invest and surround almost all tissues of the human body, and therefore cells comprising the vasculature must be capable of interacting with numerous other cell types and myriad microenvironments. The multi-cellular, multi-signalling, multi-environmental nature of new blood vessel growth therefore demands an integrated investigation of the whole system: a systems biology approach.

Angiogenesis, the growth of new blood vessels from pre-existing vessels, is an important mechanism for vascular network remodelling in the developing and adult animal. In development, initial vascular plexuses are formed by vasculogenesis, but embryos require angiogenesis during organ growth and development [1], and in adults, angiogenesis occurs in conditions requiring an increase in blood and oxygen supply, including reproduction, physiological repair (*e.g*. wound healing) and exercise [2,3]. Endothelial cells (ECs) in existing quiescent blood vessels are induced to ‘sprout’ by growth factors, and the resulting new vessel sprouts pathfind through the tissue to anastamose with existing blood vessels, forming new flow channels for oxygen delivery.

Angiogenesis is a component of several diseases, notably various cancers and retinopathies, but also arthritis, psoriasis, vascular malformations, age-related macular degeneration and primary pulmonary hypertension [4]. Uncontrolled blood vessel invasion permits the growth of tumours beyond the oxygen diffusion limit, and provides tumour cells a metastasis route. In other diseases, ectopic vascularization can cause tissue structural instability and loss of function. On the other hand, many diseases would be ameliorated by angiogenesis; these diseases have a characteristic lack or regression of blood vessels and include pre-eclampsia, Crohn's disease, atherosclerosis and osteoporosis. Therefore, we discuss how systems biology approaches can provide insight into novel or optimized therapeutics for these diseases.

Systems biology takes an integrative and holistic approach rather than a reductionist approach to understanding the mechanisms underlying a physiological process or disease. It inherently recognizes the complexity of the interconnected gene, protein, and cell pathways within tissues, and attempts to be predictive by combining quantitative and high-throughput (HTP) experimental techniques with computational models. This can identify individual targets/pathways for greatest success. Angiogenesis is an excellent example of a complex, multi-pathway, multi-cellular system that can be studied using this approach. Systems biology is greatly assisted by the inclusion of data from large-scale/HTP experiments, and in doing so we can isolate and integrate parts of the angiogenesis process in both space and time. In addition, modelling approaches can estimate experimental parameter values, losses, gains and perturbations that are difficult or impossible to measure [5]. Systems biology also permits the integration of multiple models and approaches, each appropriate for the scale or elements being studied.

## Systems biology approaches to understand the multi-step process of angiogenesis

**Fig. 1:**
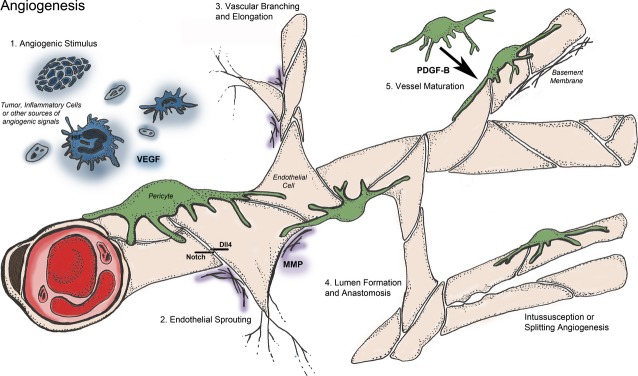
Timeline of the angiogenesis process. Angiogenesis is a morphogenesis process that consists of several discrete steps: (1) angiogenic stimulus, (2) endothelial sprouting, (3) vascular branching and elongation, (4) lumen formation and anastomosis and (5) vessel maturation. These concerted steps involve a plethora of molecular signals (Fig. 2) that carry communication between different cell types in the microenvironment, ultimately giving rise to a new blood vessel. Although sprouting angiogenesis has been studied most widely, there are other modes of angiogenesis including intussusception and vessel splitting.

Angiogenesis (neovascularization) occurs through a series of defined steps: angiogenic stimulus; sprouting; elongation and branching; lumen formation; anastomosis and finally stabilization or regression (Fig. 1). We will outline these steps and highlight published systems biology studies for each. We will discuss both pro- and anti-angiogenic cues, and the angiogenesis steps in which they are involved.

### Angiogenic stimuli

The process of angiogenesis initiates outside the bloodstream and vasculature with a distress signal from parenchymal tissue. Pro-angiogenesis scenarios include: post-vasculogenesis remodelling during organ development; wound healing; exercise and tumour growth [6]. Systems biology approaches are useful to study the commonalities and divergences in these various angiogenesis milieus and their resultant vascular networks. In all cases, the tissue signals to the existing vasculature that there is a need for new blood vessel growth. The distress signal may result from hypoxic, metabolic or mechanical stimuli [7–11] and is sensed primarily by the endothelium. For example, chronic hypoxia because of benign or malignant tissue growth, vascular occlusion or increased metabolic rates, will cause parenchymal and/or stromal cells to secrete growth factors that target the vasculature. During exercise, muscle contractions deform the vascular space leading to increased shear stress in the microcirculation and increased mechanical strain in the muscle fibres; both result in the expression of pro-angiogenic agents [12]. In all cases, angiogenesis results in an increase in tissue blood flow and tissue oxygenation.

#### Blood flow

The first experimental work relating blood flow to angiogenesis was completed in the late 1800s and early 1900s [13,14]. These studies demonstrated that blood velocity in the chick embryo, frog larvae and rabbit ear regulated both capillary growth and regression. Soon after, the first theoretical model of blood flow emerged, which related blood flow and capillary network structure to tissue oxygenation [15]. Since then, many computational models of blood flow have been developed and refined, with one of the most widely cited being that of Pries *et al*. [16]. This model acknowledges heterogeneities in the operation of the microcirculation (diameter, flow rate, shear stress and ultimately oxygen exchange), which influence not only angiogenesis but also angioadaptation or structural adaptation of the vessel wall [16]. Using experimental measurements of microvascular architecture and rheology, these simulations are able to predict stimuli and ‘rules’ for microvascular remodelling [17–19]. The predictive power of this model was demonstrated in simulations of oxygen distribution and regulation, which showed that a metabolic sensor in the vascular wall was necessary to match experimental results [20].

The models by Pries and Secomb address vascular features including vascular diameter, wall thickness and local oxygen partial pressure in a static vascular network. Other flow-based models have since incorporated vessel dynamics into simulations addressing tissue oxygenation during angiogenesis. In a model of capillary networks in tissue experiencing the high oxygen consumption rates expected during muscle exercise, sprouting angiogenesis most efficiently reoxygenated the tissue, while in simulations with equal pressure drop across the capillary bed, intussusceptive angiogenesis, which is a capillary splitting into two longitudinally and will be discussed in more detail later in this review, was slightly more efficient [21]. This study demonstrated that for a given distress signal, the flow conditions can dictate the type of angiogenic response to maximize oxygen delivery.

#### Hypoxia

Decreased blood flow can result in lower oxygen delivery (ischaemia), but changes in tissue oxygenation are not only related to blood flow. Hypoxic conditions can also result from changes in oxygen intake caused by respiratory distress or red blood cell dysfunction including anaemia [22]. Hypoxia stimulates angiogenesis through multiple pathways including nitric oxide and hypoxia-inducible transcription factors (HIFs). Both have been studied using mathematical models.

Oxygen is required for nitric oxide production from nitric oxide synthases (eNOS, nNOS, iNOS), which then feed back to inhibit tissue oxygen consumption. The interdependency between O_2_ consumption and nitric oxide regulation prompted mathematical investigation of how nitric oxide production may be regulated by the Fahraeus Effect, *i.e*. that the haematocrit observed in the microcirculation (blood vessels <200 μm) is lower than that of the macrocirculation [23,24]. This study showed that the Fahraeus Effect is most significant in blood vessels less than 30 μm in diameter, the same vessels from which angiogenic sprouting occurs. The intricate structure of the microcirculation combined with heterogeneities in the haematocrit can lead to pockets of hypoxia in the tissue that become involved in the patterning of new blood vessel growth. Tumour growth and metastasis have also been shown to be dependent on nitric oxide, because of nitric oxide-regulated angiogenesis, *via* promotion of vascular permeability and of EC proliferation and migration [25]. Although it is difficult to make experimental measurements of O_2_ transport in the microcirculation, theoretical models have shed light on critical components of this pathway. For a comprehensive review of computational models of O_2_ transport in the microcirculation, see [26] and [27]. Models of the interplay of oxygen and nitric oxide have been built [28] but have not yet been integrated into models of angiogenesis. Increased shear stress on ECs also promotes nitric oxide production, highlighting a commonality between chemical and mechanical pathways for sprouting angiogenesis [11]. Alterations in shear stress *via* muscle contractions, for example, have been directly coupled to increased eNOS and nNOS expression by skeletal muscle fibres promoting the growth of new capillaries in exercised tissues [11]. The tissue environment also plays a role in oxygen consumption and regulation, which was demonstrated by Liu *et al*. in a multi-scale model of O_2_ transport in skeletal muscle [29]. The authors modelled the influence of muscle fibre type on O_2_ distribution within the tissue and found that of the fibre properties examined (fibre size, O_2_ consumption, myoglobin concentration and oxygen diffusivity), the distribution of fibre sizes had the largest effect.

Decreased O_2_ tension promotes stabilization of HIFs, which act as transcription factors for VEGF and its receptors (VEGFRs). The VEGF family of cytokines are pro-angiogenic and have been the subject of many systems biology studies described later in this article. The best studied member of the HIF family is HIF-1 which contains two subunits: an O_2_-sensitive subunit, HIF-1α, and a constitutively active subunit HIF-1β/ARNT. In the presence of O_2_, prolyl hydroxylation and ubiquitination target HIF-1α for degradation; in low O_2_ situations HIF-1α remains stable, translocates to the nucleus and binds to HIF-1β. Here HIF-1 binds to hypoxia response elements in VEGF and VEGFR genes, up-regulating their expression. Laise *et al*. examined HIF-1-induced VEGF-A production by cancer cells with both a deterministic model containing unlimited molecular amounts and a stochastic model with finite amounts [30]. The authors showed that only the stochastic model could demonstrate cooperative behaviours between cells. For example, in extremely hypoxic environments, many cells may become apoptotic and stop production of VEGF-A. The ‘average’ response in the deterministic model was complete cell death. However, in the stochastic model, some results showed a rescue of the apoptotic cells by neighbouring cells operating in the hypoxic regime, leading to progression of a pro-angiogenesis environment. This rescue is a behaviour observed in clinical tumour growth. In the first kinetic molecular model of HIF-1α regulation [31], two behaviours were observed in HIF-1 activity depending on the microenvironment: (*i*) a switch-like mechanism, turning on all pro-angiogenic genes at a critical O_2_ level and (*ii*) a gradual mechanism resulting in a graded response. These studies show that multiple systems biology approaches are possible and may illuminate different aspects of the underlying biology.

When avascular tumours exceed a critical size (∼2 mm in diameter) they cannot be sustained by the surrounding vasculature because of the diffusion limit for oxygen [32]. At this point, the tumour cells become hypoxic and, if they have undergone appropriate transformation (‘the angiogenic switch’), secrete a host of angiogenic signals; in early literature these were lumped together and generalized as tumour angiogenic factors (TAFs). Computational modelling of TAF-induced angiogenesis began in 1985 with a model by Balding and McElwain [33]. Since that time, both continuum models (tracking densities of ECs) and discrete models (tracking explicit cells or vessels) have been used to investigate tumour growth and treatment [34–38]. In a multi-scale model, a continuum tumour growth module was coupled to a discrete angiogenesis module *via* TAF production and extracellular matrix (ECM) degradation [35,39]. This model is novel in its inclusion of blood flow and subsequent vascular diameter changes (termed ‘dynamic adaptive tumour-induced angiogenesis’ or DATIA), which result from changes in stress and pressure, and built upon work by Pries and Secomb [17]. These inclusions allow researchers to test vascular drug delivery schemes and demonstrate the clinically observed phenomena that interstitial tumour pressure blocks blood flow and increases hypoxia, leading to TAF production, angiogenesis and tumour growth. The DATIA model also allows for the investigation of vessel normalization, whereby tumour vasculature morphology changes to more closely resemble normal vessels [40,41]. For a comprehensive review of computational work modelling tumour angiogenesis, see [42–45].

#### Inflammation

During tumour angiogenesis and wound healing, hypoxia recruits bone marrow-derived cells (BMDC), including macrophages, monocytes and progenitor cells, which produce chemoattractants and other growth factors (*e.g*. VEGF) that mediate the growth of new blood vessels [46]. Unlike tumour progression, which does not have a clear end-point, wounds that heal have restored tissue oxygenation, naturally feeding back to restore BMDC numbers and the levels of angiogenic factor to normal. Continuum models of wound healing incorporating macrophage-derived signals recapitulate not only observed phenomena in wound healing (*e.g*. brush borders) but also failed wound closures (*e.g*. ulcers) [47,48]. A partial differential equation-based model by Xue *et al*. further demonstrated that ischaemia can decrease macrophage accumulation necessary for wound repair, highlighting the links and dependencies between angiogenic stimuli [48].

#### VEGF

Regardless of the initial stimulus for angiogenesis, the production of VEGF by parenchymal cells (*e.g*. BMDCs in wound repair, tumour cells in cancer progression, or skeletal muscle fibres in exercise) typically mediates the first phase of capillary growth. As mentioned previously, the VEGF family of cytokines are well-known pro-angiogenic agents in both physiological and pathological situations. It has been shown experimentally that VEGF proteins increase EC survival, proliferation and migration, where gradients guide both single cell trajectories and blood vessel patterning [49–51]. There are five VEGF ligand genes (VEGF-A, VEGF-B, VEGF-C, VEGF-D and placental growth factor, PlGF), each with unique splice variants. These ligand proteins bind to hetero- and homo-dimerized combinations of five major receptors (VEGFR1, VEGFR2, VEGFR3, and co-receptors neuropillins 1 and 2), of which there are both soluble and membrane-bound forms [50,52–54]. To add to the complexity, each VEGF ligand has a different ability to bind to the ECM, leading to immobilization in the extracellular space or free diffusion [53,55]. The complexity of VEGF signalling makes it an excellent candidate for systems biology investigation, where experimental spatio/temporal measurements of many molecular species would be impossible. Molecular models of VEGF and VEGFR expression, intra- and extracellular localization, ligand-receptor binding, and downstream signalling in ECs have all been published [56–59]. Multi-scale models have been developed that relate oxygen tension to the production of VEGF by skeletal muscle fibres during exercise and peripheral artery disease [21,56,58,60]. In one multi-scale model of skeletal muscle, exercise was predicted to be the best stimulus for increasing VEGF receptor activation and VEGF concentration gradients in ischaemia, compared to therapies that deliver exogenous VEGF, which will be discussed later. For a complete review of systems biology studies of VEGF, see [52,61].

### Sprouting

After the tissue initiates the angiogenesis cascade with a distress signal, gradients of pro- and anti-angiogenic signals develop within the tissue to guide the newly sprouting blood vessel to its final destination. In order for ECs to follow these directional cues, they must first clear a path through the vascular basement membrane and surrounding ECM. The same growth factors that guide the sprouting EC stimulate production and activation of specific proteases, which break down these structural barriers to migration. Proteases also cleave matrix-bound growth factors altering local chemotactic gradients. Selected ECs then migrate away from their host vessel up the gradient of pro-angiogenic signals (*e.g*. VEGF). The sprouting vessel is comprised of a leading ‘tip cell’ followed by a string of ‘stalk cells’ [51,62].

#### Proteases

As cells traverse the ECM, they rely on proteases to remove physical barriers to migration. One class of proteases that has been studied in the context of angiogenesis is matrix metalloproteinases (MMPs). More than 25 MMPs have been identified, each with different matrix cleavage ability and localization (diffuse or membrane-bound) [63]. Complex interactions between MMPs and other chemical species regulate activation of pro-enzymes, deactivation, degradation and secretion of these proteases. Inhibitors of MMPs include the tissue inhibitors of MMPs (TIMPS), of which TIMP-1 and TIMP-2 are expressed by ECs [63]. Detailed kinetic molecular models of MMP signalling networks have been incorporated into larger cell- and vessel-scale models [64–66]. These simulations describe the molecular events of endothelial production and activation of MMPs, as well as cellular migration. More recent models incorporate the ability of MMPs to cleave the matrix-bound VEGF isoforms (particularly VEGF_165_) from heparan sulphate proteoglycans (HSPGs) in the matrix [67,68]. In this 3D model of VEGF transport around a sprouting endothelial tip cell, Vempati *et al*. showed that protease release from the EC alone was not sufficient to cleave significant amounts of VEGF required to influence receptor signalling, demonstrating the necessity of protease production by parenchymal and/or inflammatory cells [67]. Earlier models examined MMP2 and MMP9 signalling including activation of MMP9 by MMP3 and inhibition of both MMP2 and MMP9 by TIMPs -1and -2 [64–66].

#### Concentration gradients of VEGF

Cleavage of VEGF from the matrix changes its receptor-binding capabilities as well as its local distribution. To better understand the role of protease-released VEGF during tip cell sprouting, a molecularly detailed 3D reaction-diffusion model was developed [68]. These simulations agreed with experimental observations that stronger HSPG-binding of longer VEGF isoforms (VEGF_165_ and VEGF_189_) maintains their localization in the tissue. Furthermore, the authors demonstrated that not only HSPG-binding but also isoform-specific degradation of VEGF directs sprout migration and vascular patterning during angiogenesis. ECs use filopodia to dynamically interrogate their microenvironment, sensing both diffusing and matrix-bound guidance cues (*e.g*. VEGFs, ephrins, robos and semaphorins) [51,69,70]. Only selected cells along the length of a capillary will become tip cells and begin the sprouting process. Tip cell identity and selection is based on expression of VEGFR2 and signalling through the Dll4-Notch1 system [71]. In short, VEGF binding to VEGFR2 induces expression of delta-like ligand 4 (Dll4) on an EC, which binds Notch1 expressed by the adjacent ECs. Ligation and activation of the Notch1 receptor inhibits the tip cell phenotype in these adjacent cells.

Coordination of many cells (both endothelial and parenchymal) during sprouting makes it an ideal candidate for discrete models where objects (individual cells in this case) in an environment are modelled individually, with cell behaviours defined by differential equations or logic-based rules (*e.g*., agent-based models, ABM) [72–75]. These models explicitly represent time and space, as well as the associated evolution of properties and behaviours associated with objects [76]. For example, a discrete cell-based model describing EC behaviours in tumour angiogenesis was based on the biochemical dynamics of cell–cell and cell–matrix interactions (VEGF diffusion and binding as well as matrix degradation) computed by an adjoining PDE-based continuum model [77]. In response to different VEGF gradient profiles (steep or shallow), the model displayed unique sprout morphologies (small and large diameter vessels, respectively). These results were emergent behaviours of the model. Although the different VEGF isoforms were not explicitly used to generate gradient-types in this model, the results are consistent with experimental observations regarding isoform-specific gradient-type (*i.e*. diffusible VEGF produces shallow gradients, while matrix-bound VEGF produces steep gradients) and the resulting vascular architecture [51].

In another discrete, ABM model, Dll4 and VEGFR2 expression are tracked in each cell along a simulated capillary, and are used to compute filopodia extension, migration and proliferation [78]. In this model, tip and stalk cell phenotypes were predicted to be transient and reversible, with constant competition for the tip cell position. Advancements in the model, allowing for dynamic tip cell swapping based on VEGFR2 and Notch activity, instructed experiments that validated this theory [76]. Unlike many of the single-sprout studies described above, these ABMs simulate the bloom of multiple sprouts, many initiating from the same host vessel, a phenomenon that is characteristic of angiogenesis *in vivo*. The synchronized guidance and spacing between projecting sprout tips therefore becomes important to both maintaining the function of the host capillary and responding to the metabolic demand of the distressed tissue. Experimental literature has demonstrated that a non-signalling soluble VEGFR1 (sFlt-1) is integral to sprout guidance [79,80]. A computational model of vascular morphogenesis describing VEGF diffusion and receptor binding (to soluble and membrane-bound VEGFRs) predicted that sFlt-1 secretion by sprouting ECs can sequester VEGF, altering both the local VEGF gradient and receptor signalling [81]. In these simulations, the closer two adjacent sprouts are to each other, the more likely they are to diverge in direction. For a review of systems biology investigation of sFlt-1 in angiogenesis, see [82].

### Elongation and branching

After the sprouting, EC breaches the basement membrane and begins to migrate towards the source of the angiogenic stimulus, the angiogenic ECs now make many decisions about the new vascular structure. What guides sprout extension? Will the sprout have branches, and if so, how frequently? How many sprouts are necessary to adequately compensate the distress signal? What is the source of new ECs comprising the sprouts?

Computational investigation of this phase in angiogenesis begins with models of directed endothelial migration. Although the majority of experimental and computational work in angiogenesis examines VEGF, one of the most-cited early endothelial migration models uses acidic fibroblast growth factor (aFGF or FGF-1) as the stimulus [83,84]. Here, the chemotaxis-driven migration of ECs is modelled as discrete stochastic events. Simulations revealed that aFGF increased migration speed to the same extent that it decreased directional persistence, suggesting that these behaviours may be regulated independently and giving insight into clinical models targeting cell migration. More recent models of cell migration in angiogenesis include the effects of other chemokines, substrate stiffness, and ECM components [85–89]. For example, in a model by Bauer *et al*., varying the density and orientation of ECM fibres regulated both sprout migration speed and the degree of sprout branching, indicating that these parameters may be targets for pro- or anti-angiogenic therapies [87]. A Boolean model by Bauer *et al*. examined cross-talk between angiogenesis receptor pathways (*e.g*. VEGF, integrin and cadherin) and predicted several cell phenotypes including a migrating and proliferating tip cell (‘go and grow’) that challenges the ‘go or grow’ paradigm [90,91].

Endothelial migration during angiogenesis is a concerted multi-cellular morphogenesis process. In fact, expression of vascular endothelial (VE)-cadherin, an adherens junction protein that links adjacent ECs, decreases VEGF-induced migration of ECs [92]. Glazier-Graner-Hogeweg (GGH) models, also known as cellular potts models (CPM), have been used to investigate the roles of cell–cell adhesion, cell elongation and contact inhibition of cell proliferation in EC migration during angiogenesis [36,87,93,94]. These models are lattice-based, stochastic ABM simulations where the cell membrane is mesoscopically represented, making it an ideal tool for studying changes in cell–cell adhesion and shape, both of which are technically challenging to control and interrogate experimentally. The Glazier group used a GGH model to demonstrate that vascular patterning (sprout elongation and branching) can be regulated by contact inhibition of chemotaxis or haptotaxis; they hypothesize that cell–cell adhesion blocks pseudopod extension [94].

In another ABM, cell elongation, migration and proliferation were computationally ‘knocked out’ to investigate the contribution of each cell behaviour to vascular patterning and vessel structure [95]. Simulations where knockouts were independently varied for tip and stalk ECs revealed that stalk cell proliferation is dominant in determining total vessel length, while cell elongation (a stimulus for both proliferation and migration) governed the amount of vascular branching. In addition to these EC behaviours, wall shear stress imparted by the blood stream is known to play a role in both sprout elongation and branching [96–99]. Recalling the DATIA model of tumour-induced angiogenesis described above, the authors included rules for shear stress-dependent branching in addition to TAF-induced branching. Importantly, authors note that blood flow must be present for shear stress-mediated branching to occur, thus TAF-induced branching dominates until newly formed sprouts anastomose and form lumens [39]. Simulations revealed that highly branched networks resulting from large wall shear stress values demonstrated decreased nutrient delivery to the tumour; however, sparsely branched networks failed to deliver adequate intravenous chemotherapy. It should be noted that experiments have shown that blood flow is not required for vascular branching and subsequent anastomosis [100]. In another cell-based model of tumour-induced angiogenesis, which lacks blood flow, branching spontaneously occurs based on molecular, cellular and environmental dynamics (*e.g*. VEGF gradient characteristics, proliferation rates, and ECM composition) rather than a pre-programmed probabilistic rules [77]. The authors noted that inhomogeneities in the stroma and ECM were required for branching and led to anastomosis of adjacent sprouts. This model demonstrates how molecular signalling and basic cell behaviours can inform tissue-level structures.

### Tubulogenesis, lumen formation and anastomosis

A sprouting capillary must form a connection with another vessel and develop a lumen in order to functionally carry blood. These key steps in the angiogenesis process remains the least understood with a variety of proposed mechanisms stemming mostly from the developmental biology literature.

#### Lumen formation

Mechanisms for lumen formation, or tubulogenesis, include vacuole coalescence, cell–cell junction protein rearrangement, definitions of cell polarity (apical and basal), membrane invaginations and extracelluar lumen formation [101,102]. Although many of the same molecular players appearing earlier in the angiogenesis cascade are implicated in lumen formation mechanisms (*e.g*. VE-cadherin, VEGF-A, VEGFR2 and integrins), to date there are no existing molecular models of lumen formation. This represents a potential target for the expansion of computational models. The ability to simulate the kinetics and sensitivities of each proposed lumen formation mechanism under a multitude of conditions would be helpful in understanding ideal conditions for vascular growth and may be a potential target for anti-angiogenic therapies.

#### Anastomosis

The connection of a sprouting capillary with another capillary is termed capillary anastomosis. In creating this connection, the tip cell of the sprouting capillary becomes a stalk cell, and later a quiescent cell in a stable vessel. The idea of a transient tip cell phenotype was formulated by Bentley *et al*. based on a combination of experimental and computational studies [103]. The authors developed a Spring-Agent model (an ABM where each ‘cell-agent’ is comprised of many smaller agents held together though tensions described by Hooke's Law). The novel inclusion of spring models along the cell membrane, and more specifically between cells, led to changes in cell–cell junction size, and subsequently, the number of signalling molecules present at the junction. Emergent properties arose from the model: for example, the cell–cell junction signalling through Dll4-Notch, which governs tip cell selection in sprouting, caused the tip cell to revert to a stalk cell upon anastomosis or fusion with another tip cell. Multi-scale models going beyond sprout formation and branching are starting to include anastomosis as well. In one multi-scale model, four separate modules (blood flow, oxygen delivery, VEGF transport and cell behaviour) are integrated to simulate exercise-induced angiogenesis in skeletal muscle [104]. Here, anastomosis occurs in the cell behaviour module when the filopodia of a tip cell senses another capillary or sprout within a few microns. Although the molecular detail of anastomosis formation was not addressed, the model system has the capacity to integrate anastomosis models at the molecular or cellular levels. On a technical level, this multi-scale model demonstrated that an object-oriented language can be used to integrate existing models (continuous, discrete and hybrid) built in other languages, across the many spatial and temporal scales of the angiogenesis cascade, rather than re-building in a single universal language. This study is also part of the Physiome Project, specifically the Microcirculation Physiome, where the goal is integration and knowledge-sharing between computational models of microcirculation across the globe [105,106].

#### Intussusception

In this review, we have mostly focused on sprouting angiogenesis; non-sprouting or intussusceptive angiogenesis is another path to new blood vessel formation. As the name implies, non-sprouting angiogenesis occurs without the formation of tip cells or sprouts but instead membrane pillars extend across the lumen, fusing and eventually splitting the existing vessel in half [107,108]. Intussusception was first described in 1940 by Clark and Clark [109] and named by Burri and Tarek in 1990 [110]. This mode of angiogenesis has been implicated as the dominant means of new vessel growth in animal models with therapeutic levels of VEGF overexpression [111] and animal models with chronic shear stress induced by the vasodilator prazosin [12]. Similarly, intussusception was shown to be the primary mechanism for vascular growth during the prolonged inflammatory disease colitis [112]. There are a handful of computational models of intussusception [21,24,113–117]. Most of these models simulate the microvasculature of the chick chorioallantoic membrane (CAM) and incorporate wall shear stress as the driving force for intussusception. The most recent CAM model also includes adaptations in wall stiffness, simulating the investment of support cells or pericytes and/or ECM [114]. Interestingly, these additions were necessary for simulations to predict realistic networks. In a computational model of angiogenesis in skeletal muscle, Ji *et al*. demonstrated that splitting or intussusceptive angiogenesis was more effective than sprouting angiogenesis in maintaining tissue oxygen levels during simulations of high muscle oxygen consumption [21]. However, this advantage was lost when blood flow was normalized between angiogenesis modes, suggesting that the efficiency of new blood vessel growth, and subsequent blood flow to the tissue, may differentiate between the angiogenic modes in terms of efficiency. Vessel growth *via* intussusception is quicker than *via* sprouting and produces vessels that are less leaky [118,119]. In transitioning from 2D to 3D models, the inclusion of intussusceptive angiogenesis mechanisms in conjunction with sprouting angiogenesis mechanisms may be needed to gain further insight tissue oxygenation and tumour growth.

### Stabilization/regression

As we have seen, distress signals initiate angiogenic sprouting, sprouts are guided into the tissue space where they undergo branching, lumen formation and anastomosis with other capillaries. In the final phase of angiogenesis, the newly formed, blood-carrying capillaries receive the aid of supporting mural cells or pericytes to maintain stability and escape regression. Pericytes are specialized cells found along capillaries and post-capillary venules, which, unlike higher order vessels, lack vascular smooth muscle cells (vSMCs). The role of pericytes in capillary function and angiogenesis includes regulation of EC proliferation and migration, as well as shared production of capillary basement membrane with ECs [120,121]. It is important to note that just as pericyte investment is critical for capillary stability at the end of angiogenesis, pericyte dissociation is also necessary to allow for capillary sprouting. To date, computational models including pericytes have focused on their recruitment and investment. In one ABM, pericyte recruitment was governed by gradients of EC-generated platelet-derived growth factor B (PDGF-B) and differentiation from interstitial cells was governed by contact with sprouting ECs [122]. These simulations accurately predicted angiogenic vascular structures in response to exogenous application of VEGF or increased circumferential wall strain, specifically predicting the length of capillary covered by smooth muscle α-actin-positive pericytes.

In the context of tumour growth, a multi-module algorithm was used to investigate the roles of pericytes in neovessel maturation and mature vessel destabilization [123]. The modules represented tumour growth, angiogenesis (without pericyte investment), and vessel stabilization by pericyte investment. The molecular detail of this model included VEGF, PDGF and Angiopoietins (Ang1 and Ang2). Pericytes, and other interstitial cells, express the ligands Ang1/2, which bind to the EC receptor Tie-2. Ang1 promotes vascular stabilization whereas Ang2 promotes destabilization. Interestingly, the inclusion of vessel maturation in this model resulted in significantly slower tumour growth and appeared to mimic the clinical observation of ‘dormant’ tumours.

### Quantitative high-throughput experimental approaches

Quantitative HTP experimental approaches including arrays (gene, protein and phosphorylation) have been used *ex vivo* and *in vitro* to interrogate initiation signals and offer parameters for *in silico* modelling, potential biomarkers for cancer (early-stage detection, progress and predicted outcome) and new targets for therapeutic applications [57,124,125]. For example, a HTP gene array looking at the response of renal clear cell carcinoma to hypoxia identified diversity in prolyl hydroxylase (PHD) protein family proteins, which target HIF-1α for ubiquitination [125]. Understanding this differential regulation will be important for future studies of physiological angiogenesis and in the context of tumour angiogenesis where areas of high HIF-1α protein expression have been linked to chemotherapy resistance.

Common *in vitro* angiogenesis assays (*e.g*. culture of ECs on or in Matrigel, transwell or scratch assays) investigate single or multiple behaviours of an individual cell type, ECs, including proliferation, migration, gene/protein expression and tube formation and can do so in a HTP manner. The advent of micropatterning and microfluidic techniques generated a variety of semi-high throughput *in vitro* angiogenesis assays [9,126,127]. In an elegant combination of computational and experimental microfluidic study, presented by Das *et al*., a 3D multi-scale model predicted vascular structures and angiogenic responses in a collagen gel microfluidic device [128,129]. The model shows how the characteristics of sprouts from an EC monolayer migrating into a collagen gel depend on gel stiffness and gradients of VEGF and Ang-1. By combining computational and experimental systems biology techniques, authors are able to easily validate their model and provide tools for tissue engineering of capillaries.

Beyond *in vitro* assays, there are a plethora of *in vivo* assays (*e.g*. vascular ligation, hypoxia chambers, chick CAM assays, tumour grafts), which are able to include the behaviours of many cell types including ECs, perivascular cells, vSMCs and tissue resident cells including BMDCs from haematopoietic to non-haematopoietic origin [130]. However, these assays fail in separating the mechanism of each component and are not feasible as a high-throughput tool. To combine the strengths of *in vivo* and *in vitro* techniques, recent systems biology approaches are screening pro-angiogenic and anti-angiogenic compounds using organotypic coculture or multi-culture [131–133]. Arnaoutova and Kleinman adapted a standard angiogenesis assay of EC tube formation and generated a new protocol for high-throughput analysis of angiogenic species that can be completed in 3–6 hrs. Shirinifard *et al*. recently demonstrated a 4D platform (3D plus time) that monitors blood vessel development in the zebrafish, providing detailed kinetic and dynamic cell behaviours which are critical input for computational models of angiogenesis [134]. There is no doubt that the complex timeline of events in the process of angiogenesis, including a myriad of molecular players and the orchestrated behaviours of many cell types, makes it difficult for experimental techniques to tackle. Experiments cannot simultaneously capture both the entirety of native actions (and interactions) within the system and reduce the system to isolated mechanistic pieces. For this reason, experimental systems biology approaches will always need to be paired with computational methods (including bioinformatics) and/or traditional experimental methods.

## Application of systems biology approaches to therapeutic regulation of angiogenesis

**Fig. 2:**
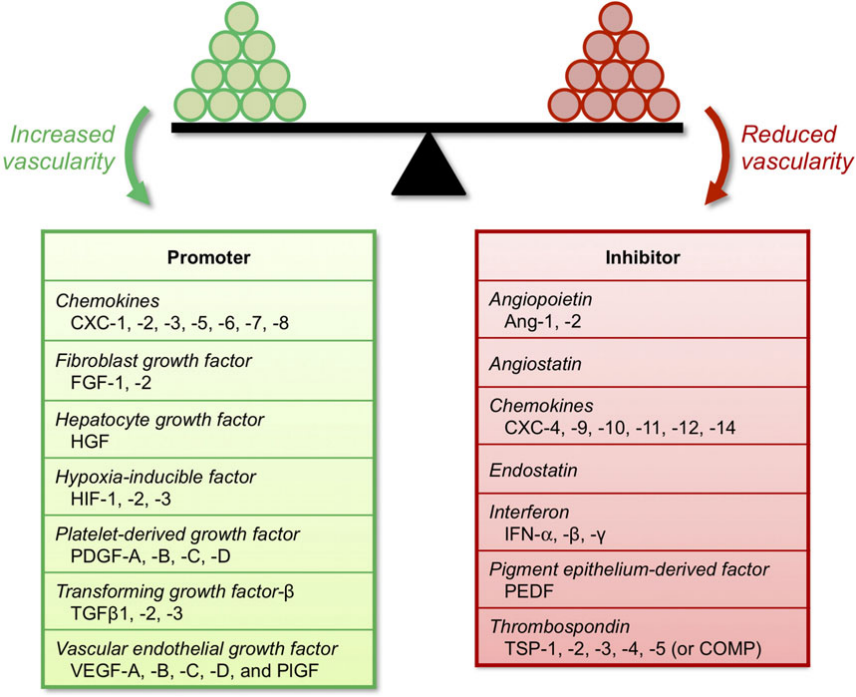
Promoters and inhibitors of angiogenesis. A balance of pro- and anti-angiogenic factors maintains homeostasis [135]. In diseases characterized by hypervascularization such as cancer and age-related macular degeneration, the balance is disrupted to promote angiogenesis. Increased expression of inhibitors of angiogenesis is a hallmark of ischaemic diseases such as coronary and peripheral arterial disease. The figure shows a short list of protein families important to the angiogenic balance, but is not exhaustive [198] and new angiogenesis modulators continue to be discovered. Many therapeutics targeting the factors named above are either already FDA-approved or are in clinical trials (see text).

As we have seen, angiogenesis is a tightly regulated, multi-step process required in many physiological conditions. A balance of pro-angiogenic factors (*e.g*. VEGF, FGF and Ang-1) and anti-angiogenic factors (*e.g*. thrombospondin-1 and endostatin) is required to maintain homeostasis in normal physiology [135] (Fig. 2). Although a shift in the relative balance of these factors must occur in response to changes in metabolic demand, for example vascular adaptation in exercise [136], an imbalance can also lead to pathological conditions. The angiogenic balance governing the extent of vascularization can be exploited in order to treat conditions for which increased vascularization is desired (*e.g*. coronary and peripheral artery diseases) or diseases characterized by hypervascularization (*e.g*. cancer and retinopathy).

Administration of exogenous factors or therapeutics can be used to control the angiogenic balance. There are already several examples of anti-angiogenic agents approved for the treatment of various forms of cancer and wet age-related macular degeneration (AMD). For example, bevacizumab (Genentech, South San Francisco, CA, USA), a recombinant humanized monoclonal antibody to VEGF, is approved for the treatment of metastatic colorectal and kidney cancer, glioblastoma and non-small cell lung cancer. Depending on the indication, bevacizumab is a monotherapy or given in combination with other drugs such as chemotherapy agents and interferon. Aflibercept (Regeneron, Tarrytown, NY, USA), a soluble fusion protein, is approved for the treatment of metastatic colorectal cancer. Pegaptanib (Pfizer, New York City, NY, USA), an RNA aptamer that specifically binds the VEGF_165_ isoform of VEGF-A, ranibizumab (Genentech), a monoclonal antibody for VEGF, and aflibercept are approved therapies to treat AMD patients. Additionally, bevacizumab is prescribed off-label to treat AMD [137]. Tyrosine kinase inhibitors (TKIs) such as pazopanib (GlaxoSmithKline, London, UK) and sunitinib (Pfizer) are approved cancer therapies and target receptors from multiple growth factor families, including receptors for FGF, PDGF and VEGF [138,139]. Other drugs are in clinical trials, such as the TKI dovitinib (Novartis, Basel, Switzerland) [140], EZN-2968 (Enzon, Piscataway, NJ, USA), an RNA antagonist that targets HIF-1α [141], ABT-510 (Abbott, Abbott Park, IL, USA), a peptide mimetic of TSP-1 [142] and AMG386 (Amgen, Thousand Oaks, CA, USA), a peptibody that neutralizes Ang-1 and -2 [143]. In contrast, no pro-angiogenic therapeutic agents have been approved, despite successes in pre-clinical animal models. For example, VEGF [144], HIF-1 [145] and FGF gene therapy [146] have failed in clinical trials [147]. However, additional pro-angiogenic therapies are in the pipeline [148]. Endogenous proteins also contribute to the regulation of angiogenesis. Platelets are a rich source of these regulatory proteins [149] and play a role in tumour angiogenesis and cancer progression [150–152]. In fact, pro- and anti-angiogenic factors are separated into specific α-granules in platelets and may be released under different conditions to promote or reduce angiogenesis [153,154]. Additionally, it has been shown that platelets sequester bevacizumab, and that the antibody can neutralize platelet VEGF [155].

The number and complexity of the events that occur during angiogenesis, along with the large number of molecular species involved, suggest that systems biology approaches are vital to the study of the process of angiogenesis. Here, we describe the use of systems biology in the development of pro- and anti-angiogenic therapies, organized according to the stages of angiogenesis presented in the previous section. Computational methods combined with quantitative experimental tools can be used to generate and validate biological hypotheses and may lead to the development of effective therapies targeting angiogenesis by predicting promising drug targets, providing a framework to test hypotheses and identifying patient population that will respond to a particular therapy.

### Targeting angiogenic stimuli

We have previously described the signals that promote angiogenesis, which include hypoxic, metabolic or mechanical cues. Given the prominent role of HIF-1 in stimulating angiogenesis and the signalling and metabolic pathways regulated by this molecular species, it is the target of numerous anti-angiogenic therapies [156–158]. For example, inhibitors of HIF-1α mRNA expression [141] and translation [159] are currently in clinical trials. Efforts to understand the HIF-1 systems have resulted in the development of several computational models [31,160–164], which can be used to identify potential therapeutic targets and predict the effects of targeting HIF-1 signalling. For example, the cellular microenvironment can promote hydroxylation of HIF-1α leading to reduced HIF-1α levels [31]. Another model predicts that PHD is able to inactivate HIF-1 and could be targeted in anti-angiogenic therapy [163]. Interestingly, recent modelling of HIF-1α signalling supported by experimental studies predicts that degradation not mediated by PHD can control the HIF-1α response during hypoxia, and asparaginyl hydroxylation provides protection from this degradation [164]. Integration of phosphoproteomics and computational modelling of insulin-like growth factor-1 (IGF-1) signalling in breast cancer was used to predict optimal drug combinations that target IGF-1 and inhibit VEGF expression [165]. One computational methodology identified cell signalling components involved in deviation from homeostasis [166]. The methodology was applied to investigate the genes that regulate the angiogenic switch (a term describing the preponderance of pro-angiogenic ligands over endogenous angiogenesis inhibitors) in pancreatic tissue, and allows for discovery of novel therapeutics that inhibit this process [166].

Computational modelling of the VEGF/VEGFR system has investigated the effects of targeting molecular species involved in this signalling pathway [167–171]. A model investigating the efficacy of targeting the VEGF co-receptor neuropilin predicted that inhibiting NRP-VEGFR coupling is a more effective strategy than blocking NRP1 expression or preventing VEGF-NRP binding [168]. Computational models are particularly strong in testing alternate hypotheses in this way. A compartment model of VEGF distribution in the body was used to investigate the mechanisms by which free VEGF in the plasma increases following anti-VEGF treatment, a counterintuitive effect that has been clinically observed [172–174]. The increase in plasma VEGF was an emergent property of the model, and could be attributed to a shuttling mechanism resulting from intercompartment transport of the antibody complexed with VEGF, *i.e*. movement of the complex between tumour, normal tissue and blood [169]. The model was expanded to include experimental quantification of VEGF receptor density, VEGF degradation and VEGF secretion by tumour cells, and a sensitivity study was performed to determine the model parameters that influence the response to anti-VEGF treatment. The resulting model predicts that upon administration of a VEGF-neutralizing agent, unbound tumour VEGF can vary depending on receptor internalization and expression on tumour cells and the specific rate of secretion of the VEGF isoforms [167,171]. The model was also applied to investigate the effect of isoform-specific anti-VEGF agents, and predicted that targeting VEGF_121_ would reduce unbound (free) VEGF in the tumour and would be an effective treatment strategy [175]. Additionally, a model of VEGF distribution in the mouse [59], validated using experimental data for VEGF Trap pharmacokinetics [176], provides a framework to explore therapies that target the VEGF pathway and can be directly compared to preclinical studies. A model of tumour growth that includes the contribution of endothelial progenitor cells (EPCs) in tumour growth was used to compare the efficacy of various anti-angiogenic therapies [170]. EPCs are bone marrow-derived cells that can localize in tumour vessels and enhance angiogenesis. The model was applied to predict the local and systemic effects of therapies targeting EC migration, VEGFR2 signalling, or the balance of pro- and anti-angiogenic factors, as well as chemo- and combination therapy. Targeting the ECs and EPCs together *via* blocking VEGFR2 signalling, restoring the balance of angiogenic factors, or combining chemotherapy and anti-angiogenic treatment, is predicted to induce systemic effects and inhibit tumour growth.

Computational models can also be applied to investigate physiological angiogenesis and pro-angiogenic therapy. A model of wound healing simulates the development of blood vessels in response to macrophage-derived angiogenesis factors [47]. The model recapitulates experimental evidence that oxygen concentration provides a feedback mechanism for vessel growth, whereby low oxygen levels influence vessel density. Oxygenation is predicted to be the dominant mechanism controlling the rate of wound healing. Another model of angiogenesis in wound healing investigates the role of macrophages and oxygen treatment to improve healing [177]. Models of VEGF distribution in skeletal muscle have been used to predict the effect of promoting VEGF signalling: up-regulation of VEGF in muscle tissue *via* cell-based or gene therapy or exercise training (*e.g*. increasing VEGFR expression) [56,58,178]. The models predict that increasing receptor expression leads to an increase in VEGF gradients and VEGFR activation for longer periods of time than up-regulation of VEGF, as well as targeting VEGFR2 signalling specifically. Thus, these models enable quantitative comparison of pro-angiogenic therapies.

### Targeting sprouting

Endothelial cells migrate into the ECM in response to pro-angiogenic stimuli. Computational models have been used to simulate the early stages of vessel sprouting and provide insight into the complex interactions of ECs [77,78,103,179,180]. Artel *et al*. combine agent-based modelling of sprouting angiogenesis in a polymer scaffold and experimental studies to investigate the relationship between neovascularization and scaffold properties [181]. Specifically, the authors vary the pore size and predict that larger pores promote faster vascularization. This study is relevant for tissue engineering applications. Another agent-based model predicts how patterns of EC behaviour such as migration, proliferation, branching and elongation lead to unique capillary sprouting in response to VEGF and brain-derived neurotrophic factor [99]. The model provides insight into how migration, proliferation, branching and elongation influence capillary structure and can aid in the development of strategies that promote vascularization in neurovascular disease. A model of blood flow in capillary networks was used to investigate the efficiency of chemotherapeutics [180] and was later extended to account for vessel pruning as a result of anti-vascular and anti-angiogenic drugs [182]. A model combining blood flow and vessel growth in tumours, termed dynamic adaptive tumour-induced angiogenesis (DATIA, described earlier), examines the effects of physical and biological parameters on the capillary network and identifies several vascular properties that are potential therapeutic targets [39]. Tip and stalk cell selection, mediated by the Notch ligand Dll-4 have been modelled [78,95,103] and complement experimental data showing that targeting Dll-4 is a promising anti-angiogenic therapy [183,184], although studies on the effects of targeting Notch/Dll-4 indicate that toxicity may prevent long-term inhibition of this pathway [185–187].

A bioinformatics approach was used to identify anti-angiogenic peptide sequences involved in inhibiting cell proliferation and migration [188]. A systematic computational analysis compared the sequences of endogenous anti-angiogenic peptides to known proteins in order to identify the common motifs and classify the peptides. Based on the analyses, several protein families were identified, including collagen type IV, CXC chemokines, somatotropins, serpins and TSP1-domain containing proteins. The ability of the peptides to inhibit proliferation and migration was validated using *in vitro* assays [188,189] and *in vivo* tumour xenografts models [190–195]. This systems biology approach revealed more than a hundred anti-angiogenic peptides that can be further studied to investigate their therapeutic potential. Additionally, Rivera *et al*. used the human interactome, which catalogues physical interactions between proteins, DNA and RNA, to identify proteins that mediate cross-talk between the families of anti-angiogenic peptides [196] and novel and missing angiogenesis annotations [197,198]. In both cases, the predictions generated through bioinformatic approaches were validated by analysis of time series gene expression data. This study illustrates that bioinformatics and related systems biology techniques have great potential utility in identifying novel targets.

### Targeting elongation and branching

Proteases are released by the sprout, both as it initiates and as it migrates, to degrade the ECM, linking the sprouting and elongation phases of angiogenesis. MMPs are a particularly well-studied family of proteases involved in angiogenesis, as described above, and they have been targeted in anti-tumour therapies. Clinical trials involving MMP inhibitors showed little therapeutic effect, in part because the inhibitors have low selectivity for specific MMPs that promote angiogenesis and tumour growth [199]. Systems biology tools can aid in the development of anti-angiogenic therapies that selectively target MMP and their substrates. For example, proteomic analysis has been shown to be a useful systems-level tool to understand the substrates of MMPs and identify potential therapeutic targets [200]. Recently, Miller *et al*. developed Proteolytic Activity Matrix Analysis (PrAMA), a novel HTP method to quantify metalloproteinase activity by combining experimental measurements and mathematical analysis [201,202]. In the first step of PrAMA, experimental fluorimetric data are obtained for several metalloproteinases against a panel of FRET-based protease substrates. The data are then fit to a kinetic model in order to determine the catalytic activity of each individual enzyme. The enzymes are clustered according to their similarity, and this serves as a ‘cleavage signature’ that can be used to infer the activity of particular enzymes in a complex biological sample. This quantitative biological information is needed to generate predictive computational models of MMP kinetics [64–66,68,203–205]. For example, a model of MMP inhibition in a multi-scale model of tumour growth [203] predicted that as cancer cells become less sensitive to anti-growth signals, the efficacy of MMP inhibition is impaired. These results may provide an explanation for the unsatisfactory results of MMP inhibitors in clinical trials, particularly since the drugs were tested in cancer patients with advanced disease. Of particular interest is a recent application of systems biology tools to predict and validate the effects of an MMP-9 inhibitor. Dufour *et al*. used *in silico* docking to identify compounds that selectively target MMP-9 and evaluated the drug candidates in biological assays and an *in vivo* breast tumour xenograft model [204]. The docking analysis revealed compounds that specifically target the haemopexin domain, a less-conserved, non-catalytic site of the MMPs, resulting in highly selective drug candidates. Based on the docking study, they identified and validated a compound that inhibits cell migration, tumour growth and metastasis induced by MMP-9. Additionally, the role of the anti-angiogenic molecule angiostatin as a protease inhibitor has been explored using mathematical models [206–208].

### Targeting tubulogenesis and anastomosis

Various steps in the angiogenesis process, including lumen formation, have been examined *via* high content screening (HCS), which uses HTP and cellular imaging techniques to obtain quantitative data on biological processes [209]. Evensen *et al*. employ HCS to visualize lumens and monitor the effects of angiogenic inhibitors [124]. ECs and vSMCs were co-cultured and treated with various anti-angiogenic and vascular disrupting agents. The total tube length, measured using live cell HTP imaging, was used to quantify the effect of the drugs. This method screens anti-angiogenic compounds in a HTP manner, enabling the identification of novel angiogenic compounds and visualization of their mode of action.

Anastomosis has been included in several models of angiogenesis [34,39,77,85,95,103,210,211]. Here, we highlight three models of particular interest. An innovative model by McDougall *et al*. combines blood flow and capillary growth and incorporates anastomosis [39]. In the model, perfusion and blood transport directly influence the formation of vascular networks, and anastomosis leads to radial adaptation and vessel remodelling. The simulated networks are examined in order to determine what parameters influence efficient transport and perfusion. The model predicts that dilated anastomoses located near a parent vessel prevent effective perfusion. Thus, targeting anastomosis and promoting the tumour vascular network to include dilated loops is a means of inhibiting the supply of blood and nutrients to the tumour [39]. As noted above, Bauer *et al*. developed the first cell-based model of tumour angiogenesis and included vessel branching and anastomosis [77]. The CPM included interactions between ECs and between an EC and its environment, including the ECM, tumour cells and interstitial fluid. Branching and anastomosis emerge based solely on cellular and molecular dynamics, as the model does not include specific rules for these morphological changes. The model predicts that branching and anastomosis are energetically favourable events that occur even in the absence of blood flow and are influenced by the composition of the stroma and ECM [77].

Models of intussusceptive angiogenesis take into account haemodynamics and biomechanical properties such as shear stress involved in vessel splitting and pillar formation [21,113,114,212]. These models are very instructive, as intussusception is a relatively new area of study compared to sprouting angiogenesis and much more research is required to gain a deeper understanding of this process [119]. Szczerba *et al*. developed a model that simulates vascular remodelling in response to haemodynamics, chemical agents and vascular wall stiffness [114]. The inclusion of secreted chemical factors (*e.g*. signalling molecules) is a novel feature of the model, which enables the prediction of the effects of modulators of intussusception under pathological conditions. Although it has not been investigated computationally, it has been shown that tumours switch from sprouting to intussusceptive angiogenesis after the administration of angiogenesis inhibitors [118].

### Targeting stabilization/regression

Pericyte recruitment and coverage promote vessel maturation and stabilization. PDGF and Ang1 are key promoters of pericyte recruitment and are prime targets for anti-angiogenic therapies. Mitchell *et al*. have developed a HTP method of quantifying pericyte coverage by measuring expression of regulator of G-protein signalling 5 (RGS5), a pericyte-specific gene [213]. Their method provides quantitative data that can be combined with efforts to model vessel maturation. A model of tumour growth and dynamics, including vessel maturation, was used to simulate anti-angiogenesis and anti-maturation therapy targeting VEGF and Ang1, respectively [123]. The model predicted that anti-VEGF therapy is more effective when the number of immature vessels is large compared to mature vessels, while anti-Ang1 treatment is not affected by this vascular property. Applying the two therapeutic agents together results in prolonged inhibition of tumour growth, as compared to single-agent therapy. The model predictions qualitatively agree with an *in vivo* model of metastatic ovarian cancer, where VEGF and PDGF are targeted with bevacizumab and a PDGF-aptamer, respectively [214]. Additionally, the development and application of sunitinib, an anti-angiogenic agent that inhibits a number of tyrosine kinases including VEGF and PDGF receptors, demonstrate the clinical relevance of combined therapies that target ECs and pericytes [139]. Similarly, a recent model of angiogenesis that simulates vessel initiation, extension and maturation, shows that inhibiting both VEGF and PDGF-B receptor β is more effective than anti-VEGF treatment alone [215]. Additionally, the model shows that vessel maturation promoted by angiopoietins and pericytes contributes to resistance to anti-VEGF therapy. Other models of tumour angiogenesis include vessel maturation and regression [216,217] and enable investigation of therapies that target this step of angiogenesis [218,219].

All of the steps involved in angiogenesis described here lead to an intricate network of vessels uniquely patterned to deliver blood and nutrients to the surrounding tissue. A model of vascular patterning predicts how the capillary network is tailored in response to parameters such as expression of angiogenic factors (both soluble and matrix-bound), MMP activity and EC proliferation rate [86]. This multi-scale phase-field model employs continuum physics, while tracking individual cells, and predicts the characteristics of the morphological features of vascular networks. Travasso *et al*. find that the diffusion properties and expression levels of the pro-angiogenic factor and the migration and proliferation rate of ECs are the primary factors that govern vascular patterning and reproduces experimental data.

## Conclusion and future work

**Fig. 3:**
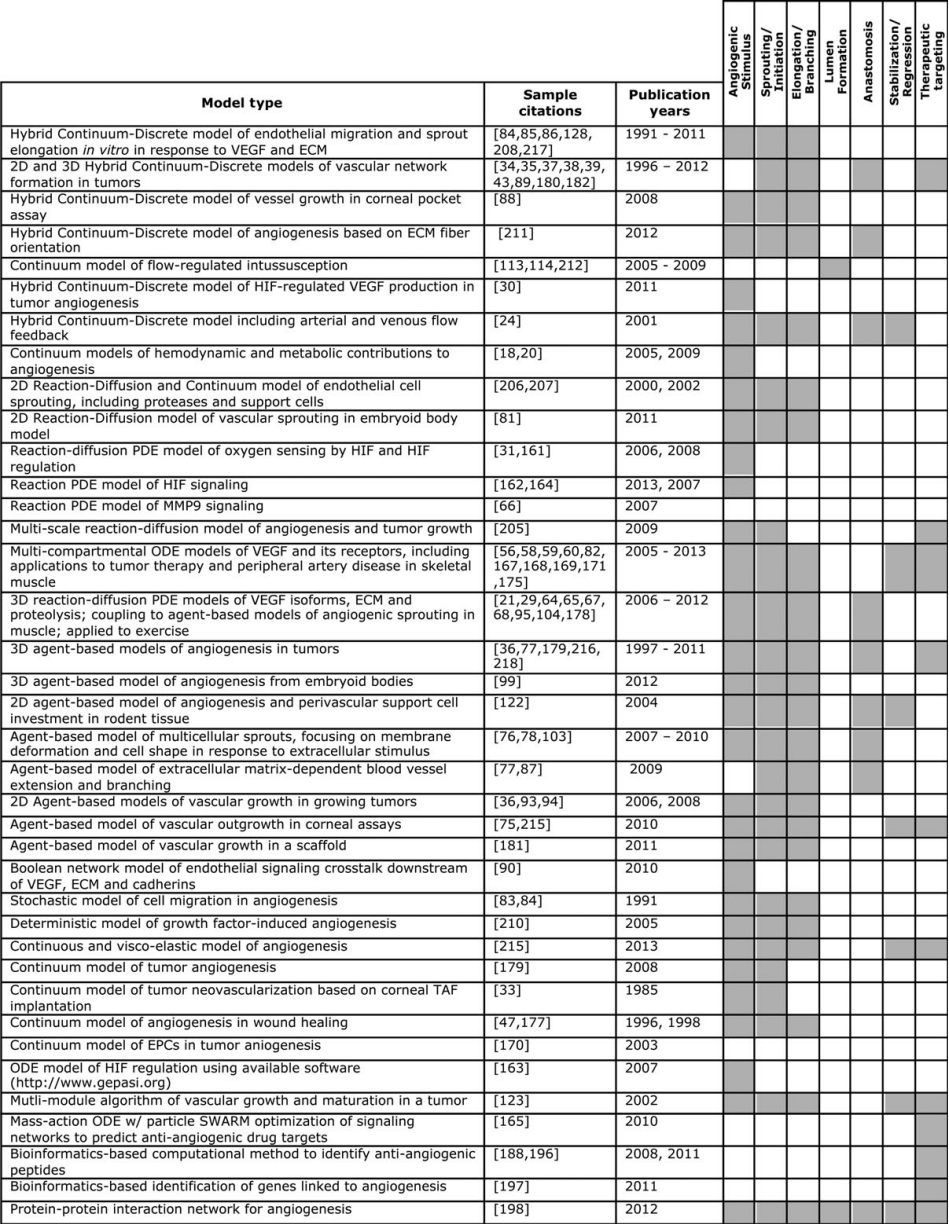
Published computational models of angiogenesis. Models of angiogenesis are collated by model type, the approximate stages of angiogenesis simulated in the models are indicated in grey and the publication years are noted. This figure displays an at-a-glance view of systems biology efforts in modelling angiogenesis.

The complexity of angiogenesis – a morphogenesis process with many stimuli, multiple steps and several cell types involved – makes its study difficult using reductionist techniques alone. Synthesizing the results of seminal experiments, incorporating data from HTP screening experiments and gene/protein arrays, and creating predictive multi-scale models (Fig. 3) are the foundations of an integrative, systems-level approach. In Figure 3, published models of angiogenesis are collated and annotated to show the part(s) of the angiogenesis process they cover. This figure visually shows that systems biology approaches to date focus on the first three steps: angiogenic stimulus, sprouting/initiation and elongation and branching. Although there are models that cover each part of the process, only a few demonstrate lumen formation, anastomosis and stabilization/regression steps. Neither the model type nor the publication year correlates with modelling of any one part of the angiogenic process; rather each part has been examined and re-examined with evolving and alternative computational methods and improved parameters. Figure 3 shows the journey of systems biology in the pursuit of angiogenesis research as well as the opportunities awaiting future modelling endeavours.

Systems biology approaches will give us the opportunity to see emergent processes and to identify, test and optimize therapeutic approaches *in silico* and *in vitro*, increasing the potential for success *in vivo*. As can be seen from this review, there remain many quantitative experimental and computational opportunities in the individual steps that comprise the angiogenesis process, as well as further gains to be made in the integration of these studies into an overarching, inclusive, mechanistic description of blood vessel remodelling.

## References

[1] Carmeliet P (2005). Angiogenesis in life, disease and medicine. Nature.

[2] Sung H-K, Michael IP, Nagy A (2010). Multifaceted role of vascular endothelial growth factor signaling in adult tissue physiology: an emerging concept with clinical implications. Curr Opin Hematol.

[3] Egginton S (2009). Invited review: activity-induced angiogenesis. Pflugers Arch.

[4] Carmeliet P (2003). Angiogenesis in health and disease. Nat Med.

[5] Bentley K, Jones M, Cruys B (2013). Predicting the future: towards symbiotic computational and experimental angiogenesis research. Exp Cell Res.

[6] Skalak TC (2005). Angiogenesis and microvascular remodeling: a brief history and future roadmap. Microcirculation.

[7] Arany Z, Foo SY, Ma Y (2008). HIF-independent regulation of VEGF and angiogenesis by the transcriptional coactivator PGC-1alpha. Nature.

[8] Fraisl P, Mazzone M, Schmidt T (2009). Regulation of angiogenesis by oxygen and metabolism. Dev Cell.

[9] Song JW, Munn LL (2011). Fluid forces control endothelial sprouting. Proc Natl Acad Sci USA.

[10] Hudlicka O, Brown M, Egginton S (1992). Angiogenesis in skeletal and cardiac muscle. Physiol Rev.

[11] Hudlicka O, Brown MD (2009). Adaptation of skeletal muscle microvasculature to increased or decreased blood flow: role of shear stress, nitric oxide and vascular endothelial growth factor. J Vasc Res.

[12] Brown MD, Hudlicka O (2003). Modulation of physiological angiogenesis in skeletal muscle by mechanical forces: involvement of VEGF and metalloproteinases. Angiogenesis.

[13] Clark ER (1918). Studies on the growth of blood-vessels in the tail of the frog larva: by observation and experiment on the living animal. Am J Anat.

[14] Thoma R (1883). Ueber die abhangigkeit der Bindegewehsneubildung in der Arterieintima von der mechanischen Bedingungen des Blutumlaufes. Arch Pathol Anat Physiol Klin Med.

[15] Krogh A (1919). The number and distribution of capillaries in muscles with calculations of the oxygen pressure head necessary for supplying the tissue. J Physiol.

[16] Pries AR, Reglin B, Secomb TW (2011). Modeling of angioadaptation: insights for vascular development. Int J Dev Biol.

[17] Pries AR, Secomb TW (2008). Modeling structural adaptation of microcirculation. Microcirculation.

[18] Pries AR, Reglin B, Secomb TW (2005). Remodeling of blood vessels: responses of diameter and wall thickness to hemodynamic and metabolic stimuli. Hypertension.

[19] Pries AR, Secomb TW, Gaehtgens P (1998). Structural adaptation and stability of microvascular networks: theory and simulations. Am J Physiol.

[20] Reglin B, Secomb TW, Pries AR (2009). Structural adaptation of microvessel diameters in response to metabolic stimuli: where are the oxygen sensors?. Am J Physiol Heart Circ Physiol.

[21] Ji JW, Tsoukias NM, Goldman D (2006). A computational model of oxygen transport in skeletal muscle for sprouting and splitting modes of angiogenesis. J Theor Biol.

[22] Varlotto J, Stevenson MA (2005). Anemia, tumor hypoxemia, and the cancer patient. Int J Radiat Oncol Biol Phys.

[23] Lamkin-Kennard KA, Buerk DG, Jaron D (2004). Interactions between NO and O_2_ in the microcirculation: a mathematical analysis. Microvasc Res.

[24] Godde R, Kurz H (2001). Structural and biophysical simulation of angiogenesis and vascular remodeling. Dev Dyn.

[25] Fukumura D, Kashiwagi S, Jain RK (2006). The role of nitric oxide in tumour progression. Nat Rev Cancer.

[26] Goldman D (2008). Theoretical models of microvascular oxygen transport to tissue. Microcirculation.

[27] Toma-Dasu I, Dasu A (2013). Modelling tumour oxygenation, reoxygenation and implications on treatment outcome. Comput Math Methods Med.

[28] Kavdia M (2011). Mathematical and computational models of oxidative and nitrosative stress. Crit Rev Biomed Eng.

[29] Liu G, Mac Gabhann F, Popel AS (2012). Effects of fiber type and size on the heterogeneity of oxygen distribution in exercising skeletal muscle. PLoS ONE.

[30] Laise P, Di Patti F, Fanelli D (2011). Deterministic and stochastic aspects of VEGF-A production and the cooperative behavior of tumoral cell colony. J Theor Biol.

[31] Qutub AA, Popel AS (2006). A computational model of intracellular oxygen sensing by hypoxia-inducible factor HIF1 alpha. J Cell Sci.

[32] Folkman J (2006). Angiogenesis. Annu Rev Med.

[33] Balding D, McElwain DL (1985). A mathematical model of tumour-induced capillary growth. J Theor Biol.

[34] Anderson AR, Chaplain MA (1998). Continuous and discrete mathematical models of tumor-induced angiogenesis. Bull Math Biol.

[35] Macklin P, McDougall S, Anderson AR (2009). Multiscale modelling and nonlinear simulation of vascular tumour growth. J Math Biol.

[36] Shirinifard A, Gens JS, Zaitlen BL (2009). 3D multi-cell simulation of tumor growth and angiogenesis. PLoS ONE.

[37] Frieboes HB, Jin F, Chuang YL (2010). Three-dimensional multispecies nonlinear tumor growth-II: tumor invasion and angiogenesis. J Theor Biol.

[38] Andasari V, Roper RT, Swat MH (2012). Integrating intracellular dynamics using CompuCell 3D and Bionetsolver: applications to multiscale modelling of cancer cell growth and invasion. PLoS ONE.

[39] McDougall SR, Anderson AR, Chaplain MA (2006). Mathematical modelling of dynamic adaptive tumour-induced angiogenesis: clinical implications and therapeutic targeting strategies. J Theor Biol.

[40] Goel S, Duda DG, Xu L (2011). Normalization of the vasculature for treatment of cancer and other diseases. Physiol Rev.

[41] Goel S, Wong AH, Jain RK (2012). Vascular normalization as a therapeutic strategy for malignant and nonmalignant disease. Cold Spring Harb Perspect Med.

[42] Stefanini MO, Qutub AA, Mac Gabhann F (2012). Computational models of VEGF-associated angiogenic processes in cancer. Math Med Biol.

[43] Chaplain MA, McDougall SR, Anderson AR (2006). Mathematical modeling of tumor-induced angiogenesis. Annu Rev Biomed Eng.

[44] Jackson TL (2012). Modeling tumor vasculature: molecular, cellular, and tissue level aspects and implications.

[45] Kim E, Stamatelos S, Cebulla J (2012). Multiscale imaging and computational modeling of blood flow in the tumor vasculature. Ann Biomed Eng.

[46] Laurent J, Touvrey C, Botta F (2011). Emerging paradigms and questions on pro-angiogenic bone marrow-derived myelomonocytic cells. Int J Dev Biol.

[47] Pettet GJ, Byrne HM, McElwain DL (1996). A model of wound-healing angiogenesis in soft tissue. Math Biosci.

[48] Xue C, Friedman A, Sen CK (2009). A mathematical model of ischemic cutaneous wounds. Proc Natl Acad Sci USA.

[49] Peirce SM, Price RJ, Skalak TC (2004). Spatial and temporal control of angiogenesis and arterialization using focal applications of VEGF164 and Ang-1. Am J Physiol Heart Circ Physiol.

[50] Tugues S, Koch S, Gualandi L (2011). Vascular endothelial growth factors and receptors: anti-angiogenic therapy in the treatment of cancer. Mol Aspects Med.

[51] Gerhardt H, Golding M, Fruttiger M (2003). VEGF guides angiogenic sprouting utilizing endothelial tip cell filopodia. J Cell Biol.

[52] Mac Gabhann F, Popel AS (2008). Systems biology of vascular endothelial growth factors. Microcirculation.

[53] Mac Gabhann F, Qutub AA, Annex BH (2010). Systems biology of pro-angiogenic therapies targeting the VEGF system. Wiley Interdiscip Rev Syst Biol Med.

[54] Koch S, Claesson-Welsh L (2012). Signal transduction by vascular endothelial growth factor receptors. Cold Spring Harb Perspect Med.

[55] Ferrara N (2010). Binding to the extracellular matrix and proteolytic processing: two key mechanisms regulating vascular endothelial growth factor action. Mol Biol Cell.

[56] Mac Gabhann F, Ji JW, Popel AS (2006). Computational model of vascular endothelial growth factor spatial distribution in muscle and pro-angiogenic cell therapy. PLoS Comput Biol.

[57] Koch S, Tugues S, Li X (2011). Signal transduction by vascular endothelial growth factor receptors. Biochem J.

[58] Mac Gabhann F, Ji JW, Popel AS (2007). Multi-scale computational models of pro-angiogenic treatments in peripheral arterial disease. Ann Biomed Eng.

[59] Yen P, Finley SD, Engel-Stefanini MO (2011). A two-compartment model of VEGF distribution in the mouse. PLoS ONE.

[60] Wu FT, Stefanini MO, Mac Gabhann F (2010). VEGF and soluble VEGF receptor-1 (sFlt-1) distributions in peripheral arterial disease: an in silico model. Am J Physiol Heart Circ Physiol.

[61] Wu FT, Stefanini MO, Mac Gabhann F (2009). Modeling of growth factor-receptor systems from molecular-level protein interaction networks to whole-body compartment models. Methods Enzymol.

[62] Blanco R, Gerhardt H (2013). VEGF and Notch in tip and stalk cell selection. Cold Spring Harb Perspect Med.

[63] Kessenbrock K, Plaks V, Werb Z (2010). Matrix metalloproteinases: regulators of the tumor microenvironment. Cell.

[64] Karagiannis ED, Popel AS (2006). Distinct modes of collagen type I proteolysis by matrix metalloproteinase (MMP) 2 and membrane type I MMP during the migration of a tip endothelial cell: insights from a computational model. J Theor Biol.

[65] Karagiannis ED, Popel AS (2004). A theoretical model of type I collagen proteolysis by matrix metalloproteinase (MMP) 2 and membrane type 1 MMP in the presence of tissue inhibitor of metalloproteinase 2. J Biol Chem.

[66] Vempati P, Karagiannis ED, Popel AS (2007). A biochemical model of matrix metalloproteinase 9 activation and inhibition. J Biol Chem.

[67] Vempati P, Mac Gabhann F, Popel AS (2010). Quantifying the proteolytic release of extracellular matrix-sequestered VEGF with a computational model. PLoS ONE.

[68] Vempati P, Popel AS, Mac Gabhann F (2011). Formation of VEGF isoform-specific spatial distributions governing angiogenesis: computational analysis. BMC Syst Biol.

[69] Hellstrom M, Phng LK, Gerhardt H (2007). VEGF and Notch signaling: the yin and yang of angiogenic sprouting. Cell Adh Migr.

[70] Klagsbrun M, Eichmann A (2005). A role for axon guidance receptors and ligands in blood vessel development and tumor angiogenesis. Cytokine Growth Factor Rev.

[71] Hellstrom M, Phng LK, Hofmann JJ (2007). Dll4 signalling through Notch1 regulates formation of tip cells during angiogenesis. Nature.

[72] Guidolin D, Rebuffat P, Albertin G (2011). Cell-oriented modeling of angiogenesis. ScientificWorldJournal.

[73] Qutub AA, Mac Gabhann F, Karagiannis ED (2009). Multiscale models of angiogenesis. IEEE Eng Med Biol Mag.

[74] Qutub AA, Mac Gabhann F, Karagiannis ED, King MR, Gee DJ (2009). In silico modeling of angiogenesis at multiple scales: from nanoscale to organ system. Multiscale modeling of particle interactions: applications in biology and nanotechnology.

[75] Jackson T, Zheng X (2010). A cell-based model of endothelial cell migration, proliferation and maturation during corneal angiogenesis. Bull Math Biol.

[76] Jakobsson L, Franco CA, Bentley K (2010). Endothelial cells dynamically compete for the tip cell position during angiogenic sprouting. Nat Cell Biol.

[77] Bauer AL, Jackson TL, Jiang Y (2007). A cell-based model exhibiting branching and anastomosis during tumor-induced angiogenesis. Biophys J.

[78] Bentley K, Gerhardt H, Bates PA (2008). Agent-based simulation of notch-mediated tip cell selection in angiogenic sprout initialisation. J Theor Biol.

[79] Chappell JC, Taylor SM, Ferrara N (2009). Local guidance of emerging vessel sprouts requires soluble Flt-1. Dev Cell.

[80] Kappas NC, Zeng G, Chappell JC (2008). The VEGF receptor Flt-1 spatially modulates Flk-1 signaling and blood vessel branching. J Cell Biol.

[81] Hashambhoy YL, Chappell JC, Peirce SM (2011). Computational modeling of interacting VEGF and soluble VEGF receptor concentration gradients. Front Physiol.

[82] Wu FT, Stefanini MO, Mac Gabhann F (2010). A systems biology perspective on sVEGFR1: its biological function, pathogenic role and therapeutic use. J Cell Mol Med.

[83] Stokes CL, Lauffenburger DA (1991). Analysis of the roles of microvessel endothelial cell random motility and chemotaxis in angiogenesis. J Theor Biol.

[84] Stokes CL, Lauffenburger DA, Williams SK (1991). Migration of individual microvessel endothelial cells: stochastic model and parameter measurement. J Cell Sci.

[85] Milde F, Bergdorf M, Koumoutsakos P (2008). A hybrid model for three-dimensional simulations of sprouting angiogenesis. Biophys J.

[86] Travasso RD, Corvera Poire E, Castro M (2011). Tumor angiogenesis and vascular patterning: a mathematical model. PLoS ONE.

[87] Bauer AL, Jackson TL, Jiang Y (2009). Topography of extracellular matrix mediates vascular morphogenesis and migration speeds in angiogenesis. PLoS Comput Biol.

[88] Tong S, Yuan F (2008). Dose response of angiogenesis to basic fibroblast growth factor in rat corneal pocket assay: II. Numerical simulations. Microvasc Res.

[89] McDougall SR, Watson MG, Devlin AH (2012). A hybrid discrete-continuum mathematical model of pattern prediction in the developing retinal vasculature. Bull Math Biol.

[90] Bauer AL, Jackson TL, Jiang Y (2010). Receptor cross-talk in angiogenesis: mapping environmental cues to cell phenotype using a stochastic, Boolean signaling network model. J Theor Biol.

[91] Giese A, Bjerkvig R, Berens ME (2003). Cost of migration: invasion of malignant gliomas and implications for treatment. J Clin Oncol.

[92] Dejana E (2004). Endothelial cell-cell junctions: happy together. Nat Rev Mol Cell Biol.

[93] Merks RM, Brodsky SV, Goligorksy MS (2006). Cell elongation is key to in silico replication of *in vitro* vasculogenesis and subsequent remodeling. Dev Biol.

[94] Merks RM, Perryn ED, Shirinifard A (2008). Contact-inhibited chemotaxis in de novo and sprouting blood-vessel growth. PLoS Comput Biol.

[95] Qutub AA, Popel AS (2009). Elongation, proliferation & migration differentiate endothelial cell phenotypes and determine capillary sprouting. BMC Syst Biol.

[96] Pries AR, Reglin B, Secomb TW (2001). Structural adaptation of microvascular networks: functional roles of adaptive responses. Am J Physiol Heart Circ Physiol.

[97] Pries AR, Reglin B, Secomb TW (2001). Structural adaptation of vascular networks: role of the pressure response. Hypertension.

[98] Long BL, Rekhi R, Abrego A (2013). Cells as state machines: cell behavior patterns arise during capillary formation as a function of BDNF and VEGF. J Theor Biol.

[99] Long BL, Rekhi R, Abrego A (2013). Cells as state machines: cell behavior patterns arise during capillary formation as a function of BDNF and VEGF. J Theor Biol.

[100] Kearney JB, Kappas NC, Ellerstrom C (2004). The VEGF receptor flt-1 (VEGFR-1) is a positive modulator of vascular sprout formation and branching morphogenesis. Blood.

[101] Herwig L, Blum Y, Krudewig A (2011). Distinct cellular mechanisms of blood vessel fusion in the zebrafish embryo. Curr Biol.

[102] Zeeb M, Strilic B, Lammert E (2010). Resolving cell-cell junctions: lumen formation in blood vessels. Curr Opin Cell Biol.

[103] Bentley K, Mariggi G, Gerhardt H (2009). Tipping the balance: robustness of tip cell selection, migration and fusion in angiogenesis. PLoS Comput Biol.

[104] Liu G, Qutub AA, Vempati P (2011). Module-based multiscale simulation of angiogenesis in skeletal muscle. Theor Biol Med Model.

[105] Bassingthwaighte JB (2008). Microcirculation and the physiome projects. Microcirculation.

[106] Popel AS, Pittman RN, Bronzino JD, Peterson DR (2013). The microcirculation physiome. The biomedical engineering handbook.

[107] Egginton S, Zhou AL, Brown MD (2001). Unorthodox angiogenesis in skeletal muscle. Cardiovasc Res.

[108] Burri PH, Hlushchuk R, Djonov V (2004). Intussusceptive angiogenesis: its emergence, its characteristics, and its significance. Dev Dyn.

[109] Clark ER, Clark EL (1940). Microscopic observations on the extra-endothelial cells of living mammalian blood vessels. Am J Anat.

[110] Burri PH, Tarek MR (1990). A novel mechanism of capillary growth in the rat pulmonary microcirculation. Anat Rec.

[111] Gianni-Barrera R, Trani M, Fontanellaz C (2013). VEGF over-expression in skeletal muscle induces angiogenesis by intussusception rather than sprouting. Angiogenesis.

[112] Konerding MA, Turhan A, Ravnic DJ (2010). Inflammation-induced intussusceptive angiogenesis in murine colitis. Anat Rec.

[113] Szczerba D, Szekely G (2005). Computational model of flow-tissue interactions in intussusceptive angiogenesis. J Theor Biol.

[114] Szczerba D, Kurz H, Szekely G (2009). A computational model of intussusceptive microvascular growth and remodeling. J Theor Biol.

[115] Lee GS, Filipovic N, Miele LF (2010). Blood flow shapes intravascular pillar geometry in the chick chorioallantoic membrane. J Angiogenes Res.

[116] Sandau K, Kurz H (1994). Modelling of vascular growth processes: a stochastic biophysical approach to embryonic angiogenesis. J Microsc.

[117] Levin M, Ewald AJ, McMahon M (2007). A model of intussusceptive angiogenesis. Novartis Found Symp.

[118] Ribatti D, Djonov V (2012). Intussusceptive microvascular growth in tumors. Cancer Lett.

[119] Styp-Rekowska B, Hlushchuk R, Pries AR (2011). Intussusceptive angiogenesis: pillars against the blood flow. Acta Physiol.

[120] Ribatti D, Nico B, Crivellato E (2011). The role of pericytes in angiogenesis. Int J Dev Biol.

[121] Gaengel K, Genove G, Armulik A (2009). Endothelial-mural cell signaling in vascular development and angiogenesis. Arterioscler Thromb Vasc Biol.

[122] Peirce SM, Van Gieson EJ, Skalak TC (2004). Multicellular simulation predicts microvascular patterning and *in silico* tissue assembly. FASEB J.

[123] Arakelyan L, Vainstein V, Agur Z (2002). A computer algorithm describing the process of vessel formation and maturation, and its use for predicting the effects of anti-angiogenic and anti-maturation therapy on vascular tumor growth. Angiogenesis.

[124] Evensen L, Micklem DR, Link W (2010). A novel imaging-based high-throughput screening approach to anti-angiogenic drug discovery. Cytometry A.

[125] Aprelikova O, Chandramouli GV, Wood M (2004). Regulation of HIF prolyl hydroxylases by hypoxia-inducible factors. J Cell Biochem.

[126] Anderson DE, Hinds MT (2011). Endothelial cell micropatterning: methods, effects, and applications. Ann Biomed Eng.

[127] Stroock AD, Fischbach C (2010). Microfluidic culture models of tumor angiogenesis. Tissue Eng Part A.

[128] Das A, Lauffenburger D, Asada H (2010). A hybrid continuum-discrete modelling approach to predict and control angiogenesis: analysis of combinatorial growth factor and matrix effects on vessel-sprouting morphology. Philos Transact A Math Phys Eng Sci.

[129] Rimchala T, Kamm RD, Lauffenburger DA (2013). Endothelial cell phenotypic behaviors cluster into dynamic state transition programs modulated by angiogenic and angiostatic cytokines. Integr Biol.

[130] Staton CA, Reed MW, Brown NJ (2009). A critical analysis of current *in vitro* and *in vivo* angiogenesis assays. Int J Exp Pathol.

[131] Arnaoutova I, Kleinman HK (2010). *In vitro* angiogenesis: endothelial cell tube formation on gelled basement membrane extract. Nat Protoc.

[132] Evensen L, Link W, Lorens JB (2010). Imaged-based high-throughput screening for anti-angiogenic drug discovery. Curr Pharm Des.

[133] Evensen L, Link W, Lorens JB (2013). Image-based high-throughput screening for inhibitors of angiogenesis. Methods Mol Biol.

[134] Shirinifard A, McCollum CW, Bolin MB (2013). 3D quantitative analyses of angiogenic sprout growth dynamics. Dev Dyn.

[135] Pollina EA, Legesse-Miller A, Haley EM (2008). Regulating the angiogenic balance in tissues. Cell Cycle.

[136] Olfert IM, Birot O (2011). Importance of anti-angiogenic factors in the regulation of skeletal muscle angiogenesis. Microcirculation.

[137] Martin DF, Maguire MG, Ying GS (2011). Ranibizumab and bevacizumab for neovascular age-related macular degeneration. N Engl J Med.

[138] Bukowski RM, Yasothan U, Kirkpatrick P (2010). Pazopanib. Nat Rev Drug Discov.

[139] Roskoski R (2007). Sunitinib: a VEGF and PDGF receptor protein kinase and angiogenesis inhibitor. Biochem Biophys Res Commun.

[140] Kim KB, Chesney J, Robinson D (2011). Phase I/II and pharmacodynamic study of dovitinib (TKI258), an inhibitor of fibroblast growth factor receptors and VEGF receptors, in patients with advanced melanoma. Clin Cancer Res.

[141] Greenberger LM, Horak ID, Filpula D (2008). A RNA antagonist of hypoxia-inducible factor-1alpha, EZN-2968, inhibits tumor cell growth. Mol Cancer Ther.

[142] Nabors LB, Fiveash JB, Markert JM (2010). A phase 1 trial of ABT-510 concurrent with standard chemoradiation for patients with newly diagnosed glioblastoma. Arch Neurol.

[143] Neal J, Wakelee H (2010). AMG-386, a selective angiopoietin-1/-2-neutralizing peptibody for the potential treatment of cancer. Curr Opin Mol Ther.

[144] Stewart DJ, Kutryk MJB, Fitchett D (2009). VEGF gene therapy fails to improve perfusion of ischemic myocardium in patients with advanced coronary disease: results of the NORTHERN Trial. Mol Ther.

[145] Creager MA, Olin JW, Belch JJ (2011). Effect of hypoxia-inducible factor-1alpha gene therapy on walking performance in patients with intermittent claudication. Circulation.

[146] AHA (2010). 2010 clinical trial/clinical science abstracts. Circulation.

[147] Mac Gabhann F, Annex BH, Popel AS (2010). Gene therapy from the perspective of systems biology. Curr Opin Mol Ther.

[148] Anghel A, Taranu G, Seclaman E (2011). Safety of vascular endothelial and hepatocyte growth factor gene therapy in patients with critical limb ischemia. Curr Neurovasc Res.

[149] Peterson JE, Zurakowski D, Italiano JE (2010). Normal ranges of angiogenesis regulatory proteins in human platelets. Am J Hematol.

[150] Bambace NM, Holmes CE (2011). The platelet contribution to cancer progression. J Thromb Haemost.

[151] Borsig L (2008). The role of platelet activation in tumor metastasis. Expert Rev Anticancer Ther.

[152] Ho-Tin-Noe B, Goerge T, Wagner DD (2009). Platelets: guardians of tumor vasculature. Cancer Res.

[153] Battinelli EM, Markens BA, Italiano JE (2011). Release of angiogenesis regulatory proteins from platelet alpha granules: modulation of physiologic and pathologic angiogenesis. Blood.

[154] Italiano JE, Richardson JL, Patel-Hett S (2008). Angiogenesis is regulated by a novel mechanism: pro- and antiangiogenic proteins are organized into separate platelet alpha granules and differentially released. Blood.

[155] Verheul HM, Lolkema MP, Qian DZ (2007). Platelets take up the monoclonal antibody bevacizumab. Clin Cancer Res.

[156] Otrock ZK, Hatoum HA, Awada AH (2009). Hypoxia-inducible factor in cancer angiogenesis: structure, regulation and clinical perspectives. Crit Rev Oncol Hematol.

[157] Park J-W, Chun Y-S, Kim M-S (2004). Hypoxia-inducible factor 1-related diseases and prospective therapeutic tools. J Pharmacol Sci.

[158] Onnis B, Rapisarda A, Melillo G (2009). Development of HIF-1 inhibitors for cancer therapy. J Cell Mol Med.

[159] Welsh S, Williams R, Kirkpatrick L (2004). Antitumor activity and pharmacodynamic properties of PX-478, an inhibitor of hypoxia-inducible factor-1a. Mol Cancer Ther.

[160] Dayan F, Monticelli M, Pouyssegur J (2009). Gene regulation in response to graded hypoxia: the non-redundant roles of the oxygen sensors PHD and FIH in the HIF pathway. J Theor Biol.

[161] Qutub AA, Popel AS (2008). Reactive oxygen species regulate hypoxia-inducible factor 1alpha differentially in cancer and ischemia. Mol Cell Biol.

[162] Yu Y, Wang G, Simha R (2007). Pathway switching explains the sharp response characteristic of hypoxia response network. PLoS Comput Biol.

[163] Yucel MA, Kurnaz IA (2007). An in silico model for HIF-a regulation and hypoxia response in tumor cells. Biotechnol Bioeng.

[164] Nguyen LK, Cavadas MA, Scholz CC (2013). A dynamic model of the hypoxia-inducible factor 1-alpha (HIF-1alpha) network. J Cell Sci.

[165] Iadevaia S, Lu Y, Morales FC (2010). Identification of optimal drug combinations targeting cellular networks: integrating phospho-proteomics and computational network analysis. Cancer Res.

[166] Hauser K, Abdollahi A, Huber PE (2009). Inverse system perturbations as a new methodology for identifying transcriptomic signaling participants in balanced biological processes. Cell Cycle.

[167] Finley SD, Engel-Stefanini MO, Imoukhuede PI (2011). Pharmacokinetics and pharmacodynamics of VEGF-neutralizing antibodies. BMC Syst Biol.

[168] Mac Gabhann F, Popel AS (2006). Targeting neuropilin-1 to inhibit VEGF signaling in cancer: comparison of therapeutic approaches. PLoS Comput Biol.

[169] Stefanini MO, Wu FT, Mac Gabhann F (2010). Increase of plasma VEGF after intravenous administration of bevacizumab is predicted by a pharmacokinetic model. Cancer Res.

[170] Stoll BR, Migliorini C, Kadambi A (2003). A mathematical model of the contribution of endothelial progenitor cells to angiogenesis in tumors: implications for antiangiogenic therapy. Blood.

[171] Finley SD, Popel AS (2013). Effect of tumor microenvironment on tumor VEGF during anti-VEGF treatment: systems biology predictions. J Nat Cancer Inst.

[172] Segerstrom L, Fuchs D, Backman U (2006). The anti-VEGF antibody bevacizumab potently reduces the growth rate of high-risk neuroblastoma xenografts. Pediatr Res.

[173] Willet CG, Boucher Y, Duda DG (2005). Surrogate markers for antiangiogenic therapy and dose-limiting toxicities for bevacizumab with radiation and chemotherapy: continued experience of a Phase I trial in rectal cancer patients. J Clin Oncol.

[174] Yang JC, Haworth L, Sherry RM (2003). A randomized trial of bevacizumab, an anti-vascular endothelial growth factor antibody, for metastatic renal cancer. N Engl J Med.

[175] Finley SD, Popel AS (2012). Predicting the effects of anti-angiogenic agents targeting specific VEGF isoforms. AAPS J.

[176] Rudge JS, Holash J, Hylton D (2007). VEGF Trap complex formation measures production rates of VEGF, providing a biomarker for predicting efficacious angiogenic blockade. Proc Natl Acad Sci USA.

[177] Schugart RC, Friedman A, Zhao R (2008). Wound angiogenesis as a function of tissue oxygen tension: a mathematical model. Proc Natl Acad Sci USA.

[178] Ji JW, Mac Gabhann F, Popel AS (2007). Skeletal muscle VEGF gradients in peripheral arterial disease: simulations of rest and exercise. Am J Physiol Heart Circ Physiol.

[179] Addison-Smith B, McElwain DLS, Maini PK (2008). A simple mechanistic model of sprout spacing in tumour-associated angiogenesis. J Theor Biol.

[180] McDougall SR, Anderson ARA, Chaplain MAJ (2002). Mathematical modelling of flow through vascular networks: implications for tumour-induced angiogenesis and chemotherapy strategies. Bull Math Biol.

[181] Artel A, Mehdizadeh H, Chiu YC (2011). An agent-based model for the investigation of neovascularization within porous scaffolds. Tissue Eng Part A.

[182] Stephanou A, McDougall SR, Anderson ARA (2005). Mathematical modelling of flow in 2D and 3D vascular networks: applications to anti-angiogenic and chemotherapeutic drug strategies. Math Comput Model.

[183] Kuhnert F, Kirshner JR, Thurston G (2011). Dll4-Notch signaling as a therapeutic target in tumor angiogenesis. Vascular Cell.

[184] Gurney A, Hoey T (2011). Anti-DLL4, a cancer therapeutic with multiple mechanisms of action. Vascular Cell.

[185] Liu Z, Turkoz A, Jackson EN (2011). Notch1 loss of heterozygosity causes vascular tumors and lethal hemorrhage in mice. J Clin Invest.

[186] Yan M (2011). Therapeutic promise and challenges of targeting DLL4/NOTCH1. Vasc Cell.

[187] Yan M, Callahan CA, Beyer JC (2010). Chronic DLL4 blockade induces vascular neoplasms. Nature.

[188] Karagiannis ED, Popel AS (2008). A systematic methodology for proteome-wide identification of peptides inhibiting the proliferation and migration of endothelial cells. Proc Natl Acad Sci USA.

[189] Koskimaki JE, Lee E, Chen W (2013). Synergy between a collagen IV mimetic peptide and a somatotropin-domain derived peptide as angiogenesis and lymphangiogenesis inhibitors. Angiogenesis.

[190] Koskimaki JE, Rosca EV, Rivera CG (2012). Serpin-derived peptides are anti-angiogenic and suppress breast tumor xenograft growth. Transl Oncol.

[191] Rosca EV, Lal B, Koskimaki JE (2012). Collagen IV and CXC chemokine derived anti-angiogenic peptides suppress glioma xenograft growth. Anticancer Drugs.

[192] Koskimaki JE, Karagiannis ED, Rosca EV (2009). Peptides derived from type IV collagen, CXC chemokines, and thrombospondin-1 domain-containing proteins inhibit neovascularization and suppress tumor growth in MDA-MB-231 breast cancer xenografts. Neoplasia.

[193] Koskimaki JE, Karagiannis ED, Tang BC (2010). Pentastatin-1, a collagen IV derived 20-mer peptide, suppresses tumor growth in a small cell lung cancer xenograft model. BMC Cancer.

[194] Lee E, Rosca EV, Pandey NB (2011). Small peptides derived from somatotropin domain-containing proteins inhibit blood and lymphatic endothelial cell proliferation, migration, adhesion and tube formation. Int J Biochem Cell Biol.

[195] Rosca EV, Koskimaki JE, Rivera CG (2011). Anti-angiogenic peptides for cancer therapeutics. Curr Pharm Biotechnol.

[196] Rivera CG, Bader JS, Popel AS (2011). Angiogenesis-associated crosstalk between collagens, CXC chemokines, and thrombospondin domain-containing proteins. Ann Biomed Eng.

[197] Rivera CG, Mellberg S, Claesson-Welsh L (2011). Analysis of VEGF-A regulated gene expression in endothelial cells to identify genes linked to angiogenesis. PLoS ONE.

[198] Chu LH, Rivera CG, Popel AS (2012). Constructing the angiome: a global angiogenesis protein interaction network. Physiol Genomics.

[199] Zucker S, Cao J (2009). Selective matrix metalloproteinase (MMP) inhibitors in cancer therapy: ready for prime time?. Cancer Biol Ther.

[200] Morrison CJ, Butler GS, Rodriguez D (2009). Matrix metalloproteinase proteomics: substrates, targets, and therapy. Curr Opin Cell Biol.

[201] Miller MA, Barkal L, Jeng K (2011). Proteolytic Activity Matrix Analysis (PrAMA) for simultaneous determination of multiple protease activities. Integr Biol.

[202] Chen CH, Miller MA, Sarkar A (2013). Multiplexed protease activity assay for low-volume clinical samples using droplet-based microfluidics and its application to endometriosis. J Am Chem Soc.

[203] Ribba B, Saut O, Colin T (2006). A multiscale mathematical model of avascular tumor growth to investigate the therapeutic benefit of anti-invasive agents. J Theor Biol.

[204] Dufour A, Sampson NS, Li J (2011). Small-molecule anticancer compounds selectively target the hemopexin domain of matrix metalloproteinase-9. Cancer Res.

[205] Billy F, Ribba B, Saut O (2009). A pharmacologically based multiscale mathematical model of angiogenesis and its use in investigating the efficacy of a new cancer treatment strategy. J Theor Biol.

[206] Levine HA, Sleeman BD, Nilsen-Hamilton M (2000). A mathematical model for the roles of pericytes and macrophages in the initiation of angiogenesis. I. The role of protease inhibitors in preventing angiogenesis. Math Biosci.

[207] Levine HA, Tucker AL, Nilsen-Hamilton M (2002). A mathematical model for the role of cell signal transduction in the initiation and inhibition of angiogenesis. Growth Factors.

[208] Plank MJ, Sleeman BD (2003). A reinforced random walk model of tumour angiogenesis and anti-angiogenic strategies. Math Med Biol.

[209] Zanella F, Lorens JB, Link W (2010). High content screening: seeing is believing. Trends Biotechnol.

[210] Sun S, Wheeler MF, Obeyeseker M (2005). A deterministic model of growth factor-induced angiogenesis. Bull Math Biol.

[211] Edgar LT, Sibole SC, Underwood CJ (2013). A computational model of *in vitro* angiogenesis based on extracellular matrix fibre orientation. Comput Methods Biomech Biomed Engin.

[212] Filipovic N, Tsuda A, Lee GS (2009). Computational flow dynamics in a geometric model of intussusceptive angiogenesis. Microvasc Res.

[213] Mitchell TS, Bradley J, Robinson GS (2008). RGS5 expression is a quantitative measure of pericyte coverage of blood vessels. Angiogenesis.

[214] Lu C, Shahzad MMK, Moreno-Smith M (2010). Targeting pericytes with a PDGF-B aptamer in human ovarian carcinoma models. Cancer Biol Ther.

[215] Zheng X, Koh GY, Jackson T (2013). A continuous model of angiogenesis: initiation, extension, and maturation of new blood vessels modulated by vascular endothelial growth factor, angiopoietins, platelet-derived growth factor-B, and pericytes. Disc Cont Dyn Syst Ser B.

[216] Owen MR, Alarcon T, Maini PK (2009). Angiogenesis and vascular remodelling in normal and cancerous tissues. J Math Biol.

[217] Plank MJ, Sleeman BD, Jones PF (2004). A mathematical model of tumour angiogenesis, regulated by vascular endothelial growth factor and the angiopoietins. J Theor Biol.

[218] Perfahl H, Byrne HM, Chen T (2011). Multiscale modelling of vascular tumour growth in 3D: the roles of domain size and boundary conditions. PLoS ONE.

[219] Honstvet CA, Jones PF (2007). Targeting tumour vasculature as a cancer treatment. Comput Math Methods Med.

